# What’s love got to do with it? Exploring social love and public health

**DOI:** 10.1177/17579139231220557

**Published:** 2024-01-25

**Authors:** RA Forbes, R Crossley, A Stevens, R James, M Black, CR Foster, E Such

**Affiliations:** School of Health and Related Research (ScHARR), The University of Sheffield, Regent Street, Sheffield S1 4DA, UK; School of Health and Related Research (ScHARR), The University of Sheffield, Sheffield, UK; Yorkshire and Humber School of Public Health, Leeds, UK; Faculty of Medicine and Health, University of Leeds, Leeds, UK; School of Health and Related Research, The University of Sheffield, Sheffield, UK; School of Health and Related Research (ScHARR), The University of Sheffield, Sheffield, UK; Anne McLaren Fellow, School of Health Sciences, Queen’s Medical Centre, University of Nottingham, Nottingham, UK

**Keywords:** social love, public health, love, health systems, systems change

## What this paper adds

- A definition of social love that addresses current professional concern over use of the concept.
- Discussion of the potential of social love to add value to public health systems, other public systems, and the populations they serve.
- A proposal of a way forward to explore the operationalisation and application of social love to existing public health decision making, and to collectively re-imagine the ways that we can incorporate ‘social love’ into everyday practice.

## Implications for policy and practice

- A public health system dominated by a biomedical model of care neglects ‘social love’, to the potential detriment of those providing and using public health services.
- Social love has the potential to add value to public health systems, other public systems, and the populations they serve, by acting as a lens through which public health policy making and decision making occurs, with the aim of improving the health and wellbeing of our populations.

## Introduction

Relationships of all types are central to our lives and to our mental and physical health.^1^ Love is an important concept in modern society, yet the popular use of the term ‘love’ does not capture the full breadth of its meaning. When one hears the word love, the mind naturally goes to romantic relationships. This presents a challenge, as the single term is used to convey several complex meanings: familial love, friendship love, parental love, spiritual love, strong individual and/or collective caring and nurturing actions towards others, and strong feelings towards objects and pastimes. The catch-all nature of the term ‘love’ is one of the primary reasons its use is often avoided by scientific, evidence-based professions, who develop their own ways of referring to the concept.^2–6^ These include using related but distinct concepts like belonging, kindness, connection, and attachment, instead of using the term love. By taking care to avoid confusion with romantic love, we, as public health professionals, are missing an opportunity to explore the impact of a concept that could be important in our practice, and in the impact that we can make in society. In this article, we explore how the concept of love may align with, enhance, and improve public health decision making and service delivery.

## Love as a Concept in Public Health and Social Care

The concept of love in health and social care is not new, and its importance in improving people’s lives is well recognised.^2–9^ Bell hooks^10^ suggested that living by a ‘love ethic’ could bring about much needed societal change, helping to overcome dominant systems of inequality, and this theory has received attention in a social care context. hooks believed that embracing a love ethic meant utilising all dimensions of love in our daily lives (care, commitment, trust, responsibility, respect, and knowledge) and taking actions that are for the collective good. hooks was not the first to describe love as action for the good of others, and there are multiple other proposed terms and slightly differing definitions of the same, including agape (the Ancient Greek term for love for everyone), compassionate love^11^, the art of loving^2^ altruistic love, tender loving care^9^, caritas^7^, unselfish love^7^, and a love of humanity^6^. hooks’ definition is of focus here for two reasons. First, hooks suggested that the ‘love ethic’ is applicable at societal, whole population level, whereas other definitions referred to here are mainly discussed in the literature in terms of individuals; and second, because it includes loving actions for the good of the self, protecting against exploitation and abuse of power^8^, whereas many of the other definitions include an element of self-sacrifice.

Another scholar, Lynn Underwood,^11^ has articulated aspects of compassionate love that overlap with hooks’ work and has developed a conceptual framework of compassionate love for use in research. Underwood describes the key features as: free choice for the other; some degree of cognitive understanding of the situation, the other, and oneself; valuing the other; openness and receptivity; and response of the ‘heart’. Underwood’s work differs from hooks’ work, in that Underwood emphasises the importance of a physical or emotional cost to self when taking compassionately loving actions (also described as a deep investment of self^12^). Despite elements of Underwood’s definition adding value to hooks’ ‘love ethic’, including valuing the other and being open and receptive, we disagree that love should include sacrifice of self and therefore align with hooks.

hooks’ work encourages a focus at a societal level, calling upon individuals to enact justice, challenge systems of power, and build connected communities. These aims are aligned with being a public health leader: having a passion to make the world a better place, advocating for traditionally excluded and disadvantaged groups, actively changing practice on the ground, working collaboratively with all stakeholders, and influencing policy at national level.^13^ Despite this, the ‘love ethic’ proposed by hooks has not yet been adequately explored in the context of public health systems, nor has it been considered beyond the individual, at organisational or institutional level. Levine and Cooney^14^ are the first authors to our knowledge to consider the potential of love and ‘generative’ relationships (where both parties are better off as a result) in transforming our lives. They suggest that ‘love, as a context within which we live, may have very powerful public health implications’ (p. 87) and describe an opportunity to consider how we ‘redesign our neighbourhoods, communities, organisations, processes, and policies to intentionally promote generative relationships, to create human systems as they were intended to be – places where it is easy to care and love one another’ (p. 88).

There are multiple reasons for the stark absence of love from the public health literature, including the prevailing biomedical model and scientisation of health, where subjectivity is less valued over objective empiricism,^4,6^ and the connotation of romance or sex from the word love. Szeintuch^3^ suggested use of the term ‘social love’ to overcome the issues around confusion of the terms ‘love ethic’ or compassionate love, with romantic or sexual love, and to ensure that the platonic nature of the love being described was immediately apparent. Szeintuch proposed a false binary however, with romantic and sexual love distinct from social love, the term under which they grouped all other types of love. We do not believe that all other forms of love can be grouped under one term. Instead, we believe that the term social love could and should be used to describe the concept created by the amalgamation of the work of hooks and Underwood outlined above.

For the purposes of this article, the term social love will be used more specifically than used by Szeintuch, to describe both the motivation and actions of a system, organisation, or institution (and people working within them), for the purpose of increasing the wellbeing of another, self, community, and the environment. Central to social love is a collective affective quality, and it involves care, respect, commitment, knowledge, responsibility, and trust, as well as valuing the other, self, community, and environment, and remaining open and receptive.

## Where does Social Love Fit in the Public Health System?

There is no single definition of a population-focused public health system, meaning it is conceptualised in different ways and often mistaken for healthcare systems that focus on the health of individuals.^15^ It can be considered as a complex system of interconnecting elements, which can promote or undermine good health and wellbeing.^16^ According to this definition, the public health system includes organisations, their underpinning policies and governance, and how they influence, work, and act within and across each other to enable or constrain actions to improve public health. In contrast many healthcare systems are rooted in a biomedical model which does not focus on wider health and wellbeing needs^17^ or love and compassion.

A public health system dominated by a biomedical model of care often neglects social love to the potential detriment of those providing and using public health services:

For those providing public health services, the academic literature suggests reasons for avoiding talking about love, including underfunding and a lack of resources, increasing technological and bureaucratic demands, and a belief that love and compassion are considered weak and unprofessional.^2,7^ Trezciak and Masserelli refer to this as a compassion crisis in healthcare that can worsen health and prolong recovery.^18^ Stickley and Freshwater argue that relationships are consistently undervalued in the UK health system and are not considered in the provision of resources,^2^ and the House of Commons Health and Social Care Committee found that health professionals are often burnt out and experience ‘compassion fatigue’.^19^

For those receiving public health services, decision making occurs at a population level, meaning there is a degree of separation between the organisation or service, and the individuals in receipt of them.^20^ This demands an awareness at organisational, service, and professional level which integrates imagination, empathy, and care, if the potential effects of population health policies and practices on an individual’s health and wellbeing are to be understood.^21^ Levine and Cooney^14^ suggest that we have unintentionally designed neighbourhoods, communities, organisations, processes, and policies that create the opposite of what is needed, because we have failed to consider the importance of generative relationships; and that the absence of generative relationships may explain our experience of entrenched and enduring health inequalities, burden of chronic disease, and poor life trajectories of so many children.

With mounting evidence that human connection and compassion is associated with the delivery of high-quality healthcare, lower healthcare costs, reduced healthcare provider burnout, and effective public health programming,^18^ now is the time to recognise the unlocked potential of social love as a concept that could introduce a new way of understanding public health policy and practice and add a new dimension to public health discourse.

## What Public Health Challenges could Social Love Help with? Violence as an Example

Violence is a serious social and public health issue, with over 1.77 million police recorded violence against the person offences in England and Wales in 2020/2021^22^ and costs to UK society estimated at over £3 billion.^23^ War and violence cost the world $14 trillion every year.^24^ The need to address the complex causes of violence is reflected in a shift towards a public health, ‘whole systems’ approach. In the diagram below, we demonstrate the potential of social love to disrupt the commonly accepted pathway to violence and outline the possible outcomes that the changes could bring. It is important to note that many of these suggestions require funding changes and decision making at central government level. Public health has always been about science and art, and this involves influencing and making the case for change.


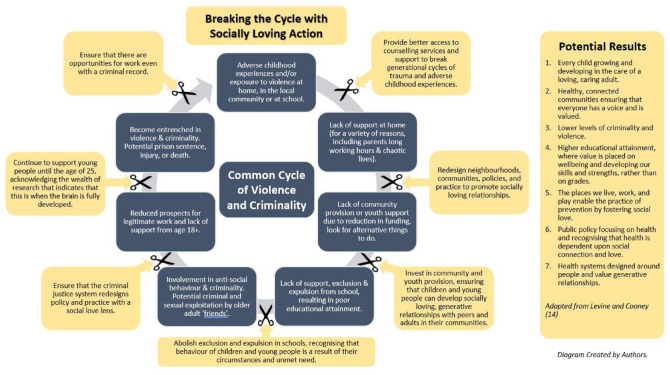


## Practically, How could Social Love Introduce a New Way of Understanding Public Health Policy and Practice, and Add a New Dimension to Public Health Discourse?

We have demonstrated in this article the potential value of social love to the health and wellbeing of the population, and that social love is currently unheard of, and potentially undervalued, in public health systems. To address these challenges, we believe it is important that people working within public health systems, other public systems, and the populations that they serve be involved in the development of the concept of social love, to understand how it relates to their purpose and core work.

## Potential Questions for the Public Health Community

We tentatively propose a ‘check and challenge’ series of questions for the public health community, as we believe it is important that organisations, services, and people working within the public health system have a starting point for considering social love in their work. Social love is a lens through which we can explore problems and solutions, and is applicable to the whole public health process, from defining a public health issue, all the way through to decision making and taking action. The definition of social love in this article provides a starting pointing for developing a more mature understanding of its potential role in public health policy and practice.

Consider each of these elements throughout the whole public health process:

Motivation: What is our ultimate aim? Is it to increase the health and wellbeing of others, self or community? This may involve asking ‘why?’ repeatedly to get to the ultimate aim.
Care: How does this impact the health, welfare, maintenance, and protection of the population/community/environment?
Respect: Are we valuing others, ourselves, our communities, and our environment? This should be regardless of circumstance, for example, even if the issue we are addressing is perceived as self-caused.
Commitment: Are we acting from a position of dedication to improving the health and wellbeing of the other, self or community?
Knowledge: Do we have an accurate understanding of the situation (the issue, the causes of it, the impacts, and unintended consequences of our proposed actions or decision), as well as the community this will affect and possible impacts to our environment?
Responsibility: Are we behaving in a socially and morally just way towards others, self, community, and environment?
Trust: Do we trust our evidence and information? Do we believe that our work is reliable/true?
Openness and receptivity: Have we been open and receptive, allowing inspiration and innovation to feature in our work?

## Next Steps

We invite interested organisations, teams, and practitioners to get in touch if they would like to explore the operationalisation and application of social love to existing public health decision making, and to collectively re-imagine the ways that we can incorporate ‘social love’ into everyday practice.
